# Blue plaque review series: Dr. Florence Buchanan: A trailblazing physiologist in an era of barriers and breakthroughs

**DOI:** 10.1113/EP092228

**Published:** 2025-04-21

**Authors:** Brian C. Clark

**Affiliations:** ^1^ Department of Biomedical Sciences, Ohio Musculoskeletal and Neurological Institute (OMNI) Heritage College of Osteopathic Medicine at Ohio University Athens Ohio USA

**Keywords:** electrophysiology, integrative physiology, muscle, neuromuscular physiology, women in science

## Abstract

What do Charles Darwin, T.H. Huxley, August Krogh and Charles Sherrington have in common? Florence Buchanan – a name history has unjustly dimmed but never erased. Her academic lineage is a direct thread through the titans of biology: E. Ray Lankester mentored her after being shaped by Huxley, Darwin's celebrated protégé. Buchanan's early career flourished under the mentorship of John Scott Burdon‐Sanderson, but it was as an independent scientist that she collaborated with Nobel laureates Krogh and Sherrington, cementing her place among the greatest physiologists of her era. While accolades marked her career – a doctor of science, prestigious fellowships and groundbreaking publications – her path was anything but easy. As the first woman to attend meetings of The Physiological Society, she broke barriers but faced systemic exclusions, barred from The Society's dinners where scientific relationships were forged. Buchanan's meticulous experiments revealed muscles’ intrinsic electrical rhythms and reshaped how physiology understands the neural control of the heart. Though her contributions were celebrated in her time, they were often overshadowed by her male colleagues. Who was this trailblazer whose scientific rigor and resilience quietly shaped modern physiology?

## Abstract (highlights)

- **What is the topic of this review?**
  This review explores the life and scientific contributions of Florence Buchanan, a pioneering physiologist whose research on muscle bioelectricity and cardiac physiology helped shape modern integrative physiology. It also examines the systemic barriers she faced as a woman in early 20th‐century science.
- **What advances does it highlight?**
  Buchanan's work provided foundational insights into neuromuscular and cardiovascular physiology, yet her contributions were often overshadowed due to gender bias. This review highlights her scientific impact, the historical inequities in academic recognition, and the broader implications for acknowledging overlooked figures in the history of science.

## 1 INTRODUCTION

In the summer of 2023, The Physiological Society reached out, inviting me to write an article for their Blue Plaque Series – a project meant to honour physiologists who have left a profound, lasting mark on the field. They specifically wanted a tribute to Dr Florence Buchanan, with a focus on how her research on mammalian reflexes and the neural control of the heartbeat laid the groundwork for integrative physiology as we know it.

My first reaction was, quite honestly, ‘Who?’ Florence Buchanan was not a name I knew. And like most mid‐career, research‐intensive faculty, I had deadlines looming – a major grant due soon and a backlog of papers to finalize. When I first saw the email, I initially assumed it was a routine solicitation. However, I was pleasantly surprised to discover it was from The Physiological Society, an organization I deeply respect. I had already started drafting my standard response – ‘Thank you, but I am overcommitted’ – when something unexpected caught my attention: they were asking me to highlight two of her papers, published in 1901 and 1908.

**FIGURE 1:**
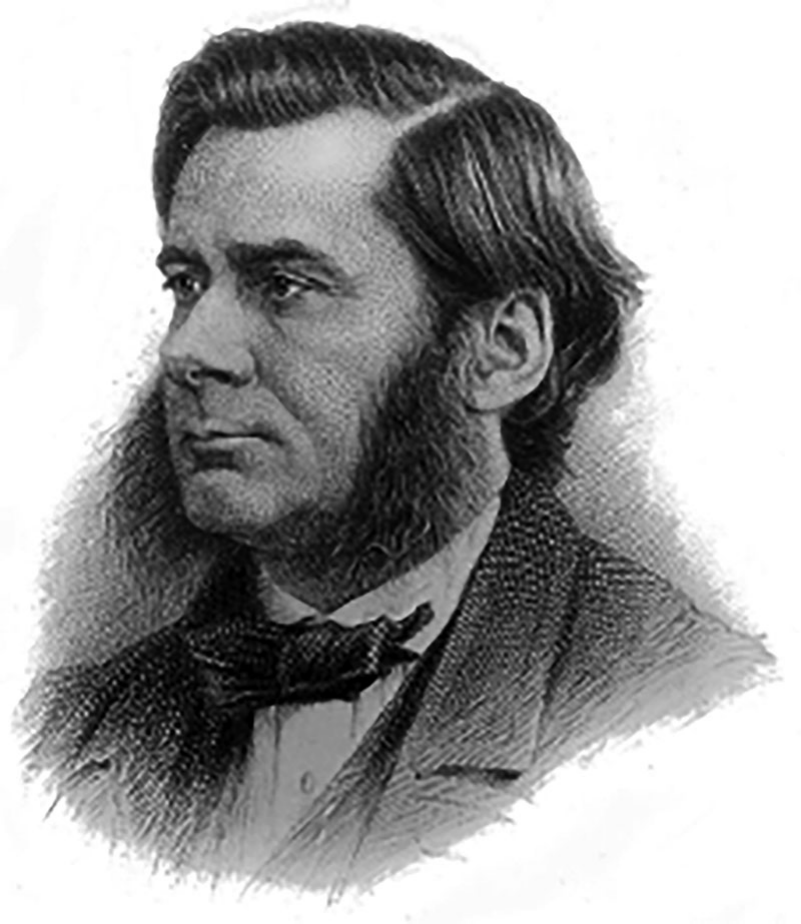
Thomas Henry Huxley (1825–1895), celebrated biologist and educator, author of *Lessons in Elementary Physiology*, a foundational textbook that introduced generations of students to the principles of human physiology. Image from Wikimedia Commons.

Suddenly, I was intrigued. Since childhood, I have been a fan of biographies. Over the years, this interest has grown into a deep respect for the history of science. That fascination began in graduate school when I stumbled upon a dusty 1915 edition of Thomas H. Huxley's (Figure 1) *Lessons in Elementary Physiology* (Huxley, 1915) in a friend's attic. It was like a time capsule; I was struck by the clarity of thought early physiologists had about the human body, despite the limited tools available to them.

After nearly three decades in science, I have witnessed astonishing technological revolutions. Today, we can sequence entire genomes in just hours using handheld devices that surpass the computational power of entire labs from a few decades ago. The rapid rise of artificial intelligence has further transformed the landscape, enabling us to analyse and interpret vast datasets with unprecedented speed and precision, opening doors to discoveries once thought impossible. Yet, the elegance and simplicity of early research remind us that foundational concepts in physiology were often established with far more basic resources. Given this context, I found myself drawn to the idea of writing about Buchanan. This was an opportunity to approach the project as a biographer, not just a scientist, and I recognized that it would challenge me to reflect on my own position – as a white male who, while not from a traditionally privileged background, has nonetheless benefited from societal structures – writing about a groundbreaking woman who defied immense odds. The challenge was as humbling as it was inspiring.

And so, I accepted the invitation – though I still had no idea where to begin. My first step was a quick internet search. A handful of brief articles and her Wikipedia page provided an outline: Florence Buchanan was a physiologist in the late 19th and early 20th centuries, primarily at Oxford, where she made significant advances in understanding heart and muscle physiology. She broke barriers, becoming one of the first women to earn a doctorate, the first woman to attend a meeting of The Physiological Society, and one of the first female members admitted in 1915.

Retrieving her papers gave me a taste of her intellect, but a deeper insight into her life was lacking. So, I circled back to The Physiological Society, asking if they could connect me with anyone who knew more about Buchanan beyond her publications. They directed me to Dame Frances Ashcroft at Oxford, and I arranged a video interview to glean any insights she might have. Though Dr Ashcroft was a rich resource in the scientific context of the era, she was quick to clarify that her knowledge of Buchanan was limited. After all, nearly half a century had passed between Buchanan's death in 1931 and Ashcroft's arrival in the department in the 1980s. Yet, it was clear that Dame Frances knew as much as anyone alive.

**FIGURE 2:**
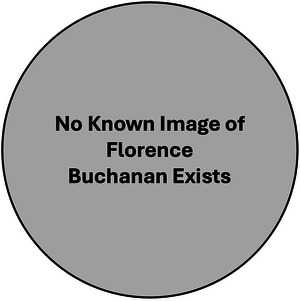
Florence Buchanan, DSc (1867–1931), pioneering physiologist, whose groundbreaking research on muscle bioelectricity and cardiac physiology contributed significantly to modern integrative physiology. Despite her remarkable achievements, no known photographs of Buchanan have been discovered, reflecting the historical erasure often faced by women in science. [Correction added on 3 June 2025 after first online publication: In the previous version, Figure 2 was potentially unclear; therefore, it has been replaced with a new figure to avoid ambiguity, and the figure caption has been accordingly updated.]

A theme was emerging: despite Buchanan's status as a trailblazer who rightly gained acclaim after her death, little of her personal story remains. No photographs or portraits of her are known to exist (Figure 2). Meanwhile, her male colleagues are remembered through photographs, biographies and even published collections of their letters, highlighting the quiet erasure that has so often overshadowed the contributions of women in science. This project, I realized, would not only be about resurrecting her science but reviving, as best I could, the memory of the woman behind it.

With these initial discoveries, I found myself drawn not only to Buchanan's scientific achievements but also to the elusive details of her life within – and often in defiance of – the constraints of her era. Who was this trailblazer whose work quietly shaped modern physiology, yet whose personal story remains so fragmented? Despite limited sources, I aimed to reconstruct a picture of Buchanan as both a scientist and a person, to shed light on the legacy of a woman whose impact far outstripped the traces left behind.

## 2 BREAKING BARRIERS

Science often celebrates those ‘eureka moments’ – breakthroughs that shift paradigms and redefine fields. But these moments are rarely as they seem, and many stories of true trailblazers remain untold. Florence Buchanan's story is one of these – a blend of innovative research, relentless determination, and a fight for recognition in an era when the field scarcely made room for her. In the late 1800s and early 1900s, society was just stepping into modernity: automobiles were rolling onto the streets, electricity was lighting up homes and inventions like the telephone were reshaping daily life. Yet, despite Buchanan's groundbreaking work in muscle and heart physiology, she faced deeply entrenched societal barriers merely to secure a seat at the table – a seat that often vanished once dinner was served (but more on that later).

## 3 FOUNDATIONS OF A LEGACY

**FIGURE 3:**
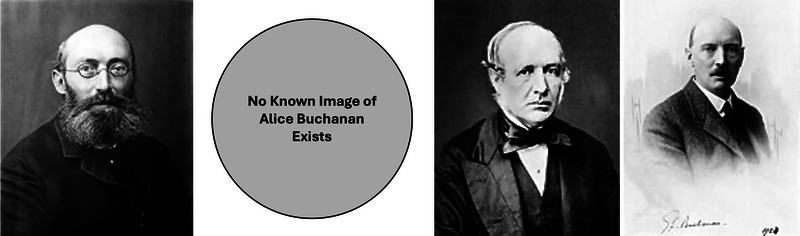
The Buchanan family legacy. A four‐panel depiction of Florence Buchanan's family, highlighting their significant contributions to public health and science. From left to right: Sir George Buchanan, MD (1831–1895): Florence's father, the United Kingdom's 3rd chief medical officer, renowned for his work in sanitation reform and disease prevention. Alice Mary Asmar Buchanan (dates unknown): Florence's mother and the daughter of Dr Edward Cator Seaton. She likely played a central role in raising Florence and her siblings in an intellectually vibrant household (no image available). Dr Edward Cator Seaton, MD (1815–1869): Florence's maternal grandfather and predecessor to Sir George as the 2nd chief medical officer, recognized for his early leadership in vaccination programs and public health. Sir George Seaton Buchanan, MD (1869–1936): Florence's younger brother, a distinguished epidemiologist whose work in disease surveillance and control advanced the field of public health. This family legacy laid the foundation for Florence's own groundbreaking career in physiology. Images from Wikimedia Commons. [Correction added on 3 June 2025 after first online publication: In the previous version, Figure 3 was potentially unclear; therefore, it has been replaced with a new figure to avoid ambiguity, and the figure caption has been accordingly updated.]

Florence Buchanan's life and career straddled a fine line between privilege and struggle. Born in 1867 into the echelons of England's upper class, she was the daughter of Sir George Buchanan (Figure 3), the United Kingdom's chief medical officer, and Alice Mary Asmar (Figure 3), whose family also boasted a remarkable legacy in public health (British Medical Journal, 1936; https://en.wikipedia.org/wiki/George_Buchanan_(physician)). Sir George was a towering figure, renowned for his contributions to sanitation reform and the prevention of infectious diseases during a transformative period in Britain's history. He succeeded Florence's maternal grandfather, Dr Edward Cator Seaton (Figure 3), in the role of chief medical officer – a position akin to the US Surgeon General, though with a broader remit to design and implement national health strategies. Florence grew up immersed in a family legacy that helped shape modern public health.

Her parents had only been married for 2 years when Florence was born. Sir George, at the time 36 years old, had been previously widowed, while Lady Buchanan was in her twenties. Florence was one of six children in the family – four daughters and two sons – who grew up in what must have been a dynamic and intellectually stimulating household. However, Florence's early adult years were also marked by profound loss. In 1895, when Florence was just 28 years old, her father passed away at 63 years of age following cardiac surgery. Losing such an influential and meaningful figure so early in her adult life may have deeply shaped her personal and professional trajectory.

Despite this legacy, Florence's name is often conspicuously absent from historical accounts, even in places where her siblings are celebrated. It is a reminder of how easily women's achievements can be overlooked.

Little is documented about Florence's early years, but the Buchanan household must have been a lively one, steeped in intellectual rigor and public service. Lady Buchanan, as the daughter of one chief medical officer and the wife of another, likely would have instilled a strong sense of discipline and ambition in her children. This dynamic no doubt shaped Florence, who chose a path different from her political and public health‐minded father and brother, Sir George Seaton Buchanan (Figure 3), a renowned epidemiologist who was appointed to several Royal Commissions. While her siblings found their own niches in the family tradition of service, Florence defied societal norms to forge a career in physiology – a field with immense barriers for women.

This duality – privilege paired with constraint – might explain Florence's determination to excel in science against the odds. Though details of her early life are sparse, it is evident that the strong intellectual foundation she built at home played a crucial role in shaping her groundbreaking work in physiology.

## 4 FORGING HER PATH

**FIGURE 4:**
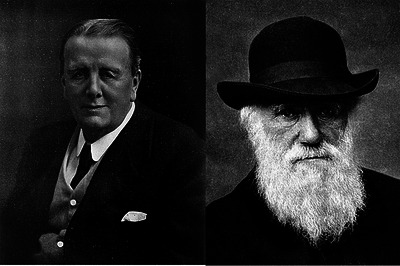
Intellectual legacy: from Darwin to Lankester. A two‐panel depiction highlighting the intellectual lineage that shaped Florence Buchanan's early scientific career. Left: Sir Edwin Ray Lankester, DSc (1847–1929): Eminent biologist and comparative anatomist, Lankester was Buchanan's mentor during her undergraduate studies at University College London. A staunch advocate of evolutionary biology, Lankester was influenced by his own mentors, including Thomas Henry Huxley. Right: Charles Darwin (1809–1882): Revolutionary naturalist and author of *On the Origin of Species*. Darwin's work laid the foundation for evolutionary biology. This photograph, taken a year before his death in 1882, captures the scientist whose ideas influenced generations, including Thomas Henry Huxley and later Lankester, shaping the intellectual environment that likely guided Buchanan's early career. This figure highlights the enduring influence of evolutionary thought on Buchanan's scientific development, as well as that of other physiologists of her time. Images from Wikimedia Commons.

Despite her privileged upbringing, Buchanan faced the deeply entrenched gender barriers of her time, which kept women from fully participating in the professional and scientific worlds. Yet, she was determined to defy these limitations. She began her journey at University College London, where, in 1890 shortly after her 23rd birthday, she received her undergraduate degree (Creese, 2004). As an undergraduate, she worked with the esteemed biologist Sir Edwin Ray Lankester, who was one of the few men at the graveside of Karl Marx when he was laid to rest at Highgate Cemetery, (Figure 4) and published at least one paper during this time. Her 1889 paper examined the respiratory systems of decapod crustaceans, such as crayfish and crabs (Buchanan, 1889). Her obituary suggests that she published multiple papers as an undergraduate (Nature, 1931), but gaps in historical records make it difficult to identify these with certainty. Regardless, publishing even a single scientific article at this stage – especially as a woman in the late 19th century – was an extraordinary accomplishment. Buchanan's path was set – she would pursue science, regardless of society's constraints.

After graduation, Florence secured a research position and continued working with Lankester, a role she held from around 1890 to 1894 (Ashcroft, 2020). Lankester, who was about 20 years her senior, was no ordinary mentor. His academic roots traced directly to two of the most influential scientists of the 19th century: he had studied under Thomas H. Huxley, the ‘Bulldog of Darwin,’ who fiercely defended evolutionary theory, and Huxley himself had been mentored by none other than Charles Darwin (Figure 4). Lankester's approach to biology was deeply rooted in evolutionary perspectives, which strongly influenced Buchanan's early research.

By the time Florence joined him, Lankester was a prominent individual in biology, serving as President of the Marine Biological Association and Professor of Comparative Anatomy at Oxford. He would later become the director of the Natural History Museum in London (https://en.wikipedia.org/wiki/Ray_Lankester; Milner, 1999). Working under his guidance not only refined Buchanan's skills as a scientist but also opened doors to invaluable connections within the elite scientific circles of Britain and beyond.

**FIGURE 5:**
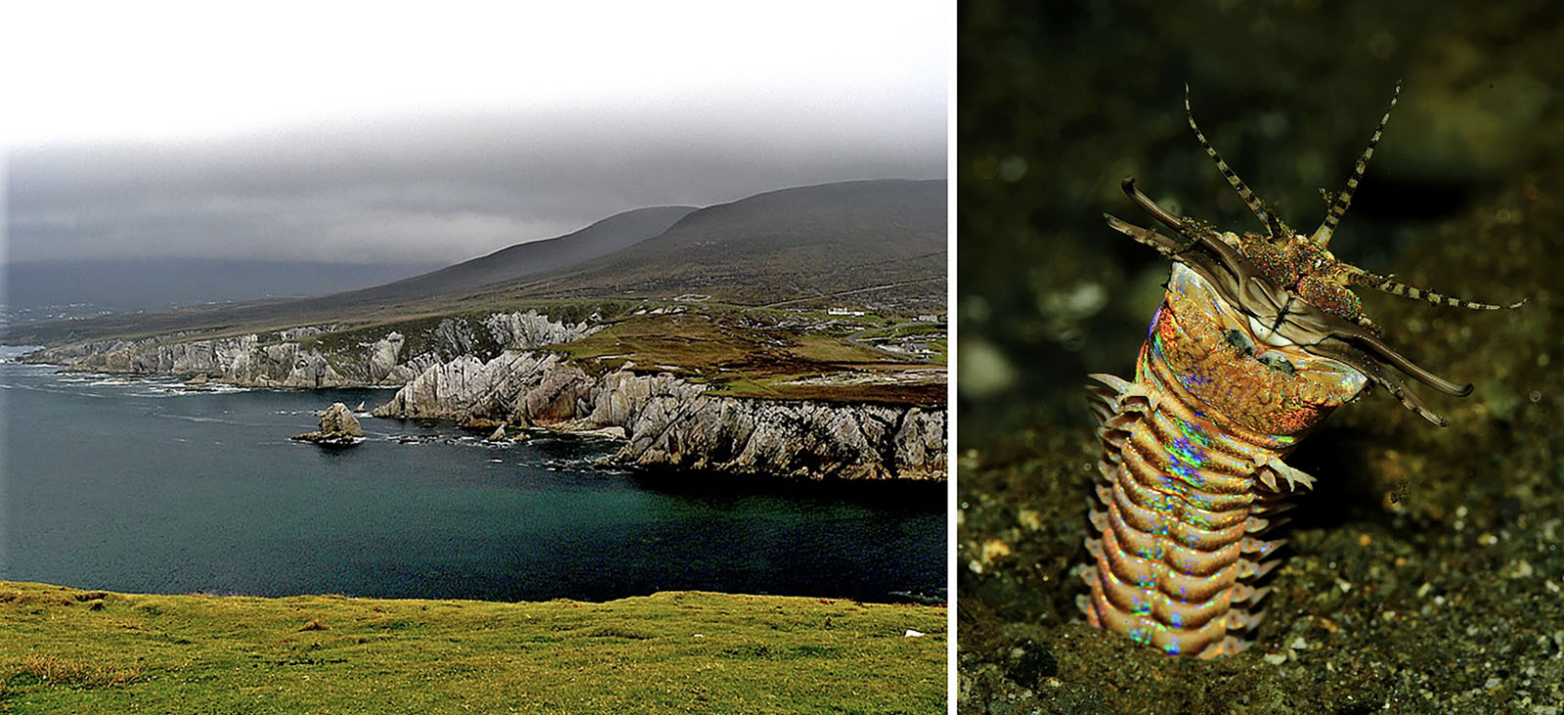
Coastal dredging and species discovery. A two‐panel figure highlighting the environments and organisms central to Florence Buchanan's early research in marine biology. Left: The wild coastline of northwest Mayo, Ireland, where Buchanan worked with Sir Edwin Ray Lankester in her mid‐20s, dredging for new marine species. Right: A photograph of *Eunice aphroditois*, a member of the polychaete family Eunicidae. While Buchanan discovered a new species in this genus, this image does not depict her specific discovery. One can imagine the thrill of these discoveries for a young scientist, immersed in the beauty of the Mayo coastline and uncovering the secrets of marine life. Images from Wikimedia Commons.

In her role as a marine biologist, Florence discovered several new species of segmented worms dredged from the waters off the Cornish and Irish coasts (Buchanan, 1893, 1894). One can picture the thrill of these discoveries and the transformative experience this must have been for a scientist in her mid‐20s, immersed in the beauty of these marine environments (Figure 5). However, Lankester himself was a complex figure. Known for his brusque manner (Huxley, 1970), he was entangled in scandal shortly after Florence moved on in her career: on 7 October 1895 he was charged with disorderly conduct and resisting arrest while having been intoxicated and ‘in the company of six or seven prostitutes’ on the street (though he was later acquitted) (Birmigham Daily Post, 1895). Indeed, Florence's formative years were shaped in a world where even scientific brilliance was often tangled with darker complexities.

## 5 PHYSIOLOGY IN TRANSITION: A NEW ERA BEGINS

**FIGURE 6:**
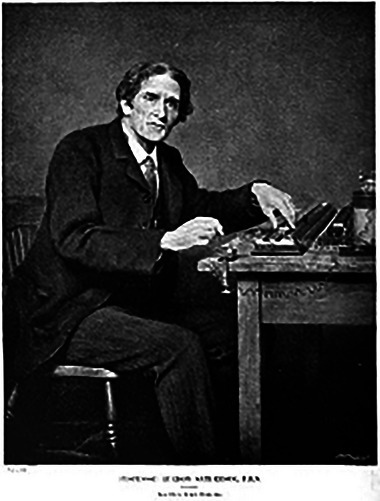
John Scott Burdon‐Sanderson, MD (1828–1905). This engraving of John Scott Burdon‐Sanderson, MD, FRS, is based on a portrait by John Collier, one of the most prominent portrait painters of his generation. He also did portraits of Huxley, Darwin, and Michael Foster in the NPG. His greatest painting is ‘Lilith’ in Southport Art Gallery. This image depicts Burdon‐Sanderson during his tenure as the Waynflete Professor of Physiology at the University of Oxford. Burdon‐Sanderson was instrumental in establishing experimental medicine in the United Kingdom. His groundbreaking work on electrophysiology and muscle function influenced generations of scientists, including Florence Buchanan, whom he mentored during her early career. This engraving underscores his significant role in shaping modern physiology. Image from Wikimedia Commons.

It was 1894 (or possibly 1895, I found some discrepancies in historical records) when at age 66 John Scott Burdon‐Sanderson (Figure 6) was appointed the prestigious Regius Professor of Medicine at Oxford (MacNalty, 1954). Burdon‐Sanderson was a well‐renowned, physiologist and pathologist whose work had been instrumental in establishing experimental, lab‐based approaches in medicine. In particular, he was known for his work in electrophysiology, muscle and nerve physiology, and infectious diseases like tuberculosis (MacNalty, 1954; Rogers, 1969). Despite facing fierce opposition from anti‐vivisectionists for his advocacy of animal research, he advanced groundbreaking discoveries in bioelectric signals of muscles and nerves using capillary electrometers (MacNalty, 1954; Rogers, 1969) – allowing him to quantify nerve impulses and muscle contractions with remarkable precision for his time.

In his prior role as Oxford's first Waynflete Professor of Physiology, Burdon‐Sanderson solidified physiology as an academic discipline and mentored many future leaders. Thus, it is no surprise that Florence Buchanan likely jumped at the opportunity to join his laboratory when Burdon‐Sanderson extended an invitation, noting she was ‘a daughter of his old friend, Sir George Buchanan’ (Burdon Sanderson et al., 1911). This position allowed Florence to immerse herself in electrophysiological studies and enter the inner circle of Britain's foremost physiologists at the turn of the century. Her relationship with Burdon‐Sanderson – as mentor, colleague and family friend – would prove pivotal, providing her with the foundation to pursue her groundbreaking work. Around 1894, the senior scientist Burdon‐Sanderson and junior scientist Buchanan began a collaboration that would span the next decade until Burdon‐Sanderson's retirement in 1904, shortly before he died in 1905 (Ashcroft, 2020).

**FIGURE 7:**
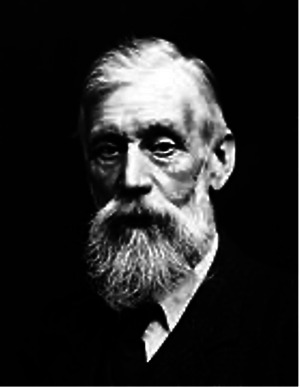
Sir Michael Foster, MD, FRS (1836–1907) was a prominent physiologist and one of the leading figures in the development of modern physiology in Britain. As the first professor of physiology at the University of Cambridge, Foster played a pivotal role in shaping the discipline, not only through his research but also as an influential educator and mentor. His contributions extended to co‐founding *The Journal of Physiology* in 1878, which remains one of the premier journals in the field today. Image from Wikimedia Commons.

Two years after joining Burdon‐Sanderson's lab, in 1896, Buchanan made history as the first woman to attend a meeting of The Physiological Society – an organization that, until that moment, had been exclusively male (Groll, 2015). Founded 20 years earlier in 1876 by Burdon‐Sanderson and Sir Michael Foster (Figure 7), who would soon launch *The Journal of Physiology*, The Physiological Society was initially established as a response to intensifying opposition to animal research, including new restrictions under the Cruelty to Animals Act of 1876 (NAVS; Groll, 2015).

In its early days, The Physiological Society functioned more like a private social club than a professional organization (Groll, 2015). Its rules allowed only 40 physiologists to gather for dinners, often held at Burdon‐Sanderson's home (Groll, 2015). For the first decade, these meetings were mainly social (Sharpey‐Schafer, 1927). By the 1880s, scientific presentations had become a regular feature, signalling a shift toward a more formal society. Yet, even as the Society evolved, its core rituals – including the dinners, where networking and discussions thrived – remained staunchly male‐only, leaving women like Buchanan on the outskirts.

While Buchanan's attendance at these meetings beginning in the late 1890s was a significant step, she was not admitted as a member, nor was she widely welcomed by many in attendance. Her presence highlighted the Society's unspoken boundaries: though she was permitted in the room, she was not fully included. For instance, while Florence was occasionally allowed to attend meetings and present her work, she was excluded from the central event of the meeting: the dinner that followed (Ashcroft, 2020; Groll, 2015). Simply attending required not only a scientific drive but also a quiet defiance, a willingness to confront the rigid gender expectations of her era. Buchanan's persistence would eventually pave the way for greater inclusivity – a legacy that will be explored further in the context of her later contributions and the evolving role of women in science.

## 6 CONTRIBUTIONS TO MUSCULAR PHYSIOLOGY

Piecing together the exact steps Buchanan took during her time working with Burdon‐Sanderson is no simple task. What we do know is this: between approximately 1895 and 1902, she conducted a series of meticulous and groundbreaking experiments, culminating in a sweeping 74‐page article published in *The Journal of Physiology* in 1901 (Buchanan, 1901). Titled ‘The electrical response of muscle in different kinds of persistent contraction’, the paper stands as a testament to her dedication, ambition and scientific rigor. Her 1931 obituary in *Nature* highlights the recognition this work earned her, including several prestigious awards, a doctor of science (DSc) degree from the University of London, and a fellowship at University College London (Nature, 1931).

Understanding the significance of her achievements requires some historical context. University College London was among the first institutions to admit women on equal footing with men (Anderson, 2006), thanks in part to advocates like Florence's father, a fellow and council member of the university, who helped to first secure their admission. Oxford, however, lagged behind; although it allowed women to attend lectures and sit for exams, it did not grant them degrees until 1920 – nearly a quarter‐century after Buchanan completed her research there (Anderson, 2006).

This split reveals much about the academic climate of the time. While Buchanan conducted her groundbreaking research in Oxford's laboratories, her official recognition – her doctorate and subsequent fellowship – came from University College London. As Dame Frances Ashcroft explained, this arrangement reflected the opportunities and limitations imposed by the differing policies of these institutions.

Buchanan's ability to navigate these divides speaks to her tenacity and adaptability, traits that shine through in her work. Her ambitious 74‐page paper, with its meticulous experimentation and profound insights, not only brought her well‐deserved acclaim but also underscored the challenges and triumphs of a pioneering woman in science. Now, let us delve into the heart of her research, the work that solidified her place in the history of physiology.

## 7 BUCHANAN'S INVESTIGATION OF ELECTRICAL RESPONSES IN MUSCLE

**FIGURE 8:**
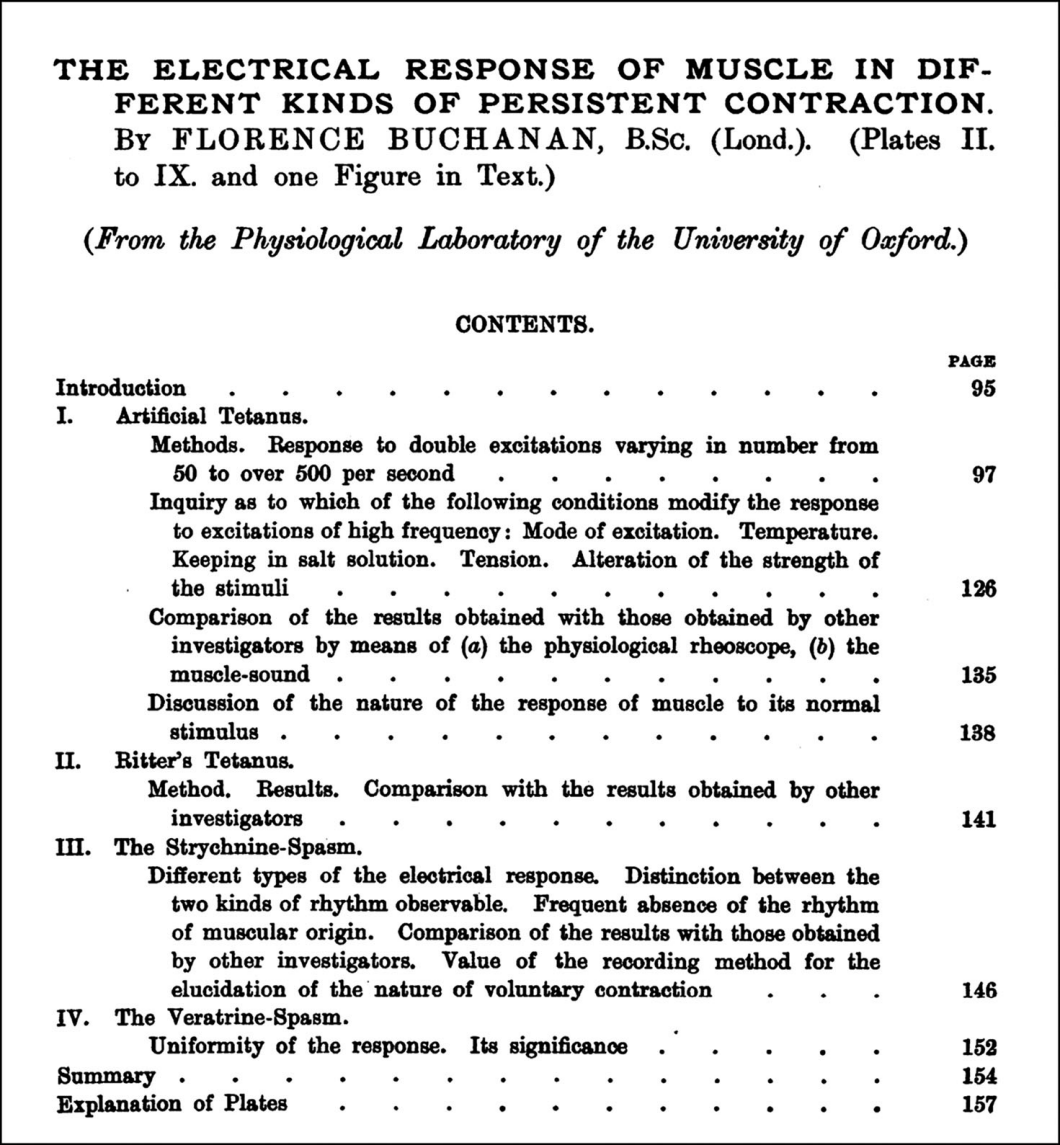
The title page of Florence Buchanan's, 1901 *Journal of Physiology* paper that exemplifies her meticulous experimental approach and contributions to early electrophysiological research. Reprinted with permission.

Florence Buchanan's 1901 paper, ‘The electrical response of muscle in different kinds of persistent contraction’ (Figure 8) (Buchanan, 1901), examined the electrical activity of skeletal muscle during various types of sustained contractions. Working with the sartorius muscle of the frog, she systematically assessed how different frequencies of stimulation, mechanical tension and chemical environments influenced muscle electrical responses.

**FIGURE 9:**
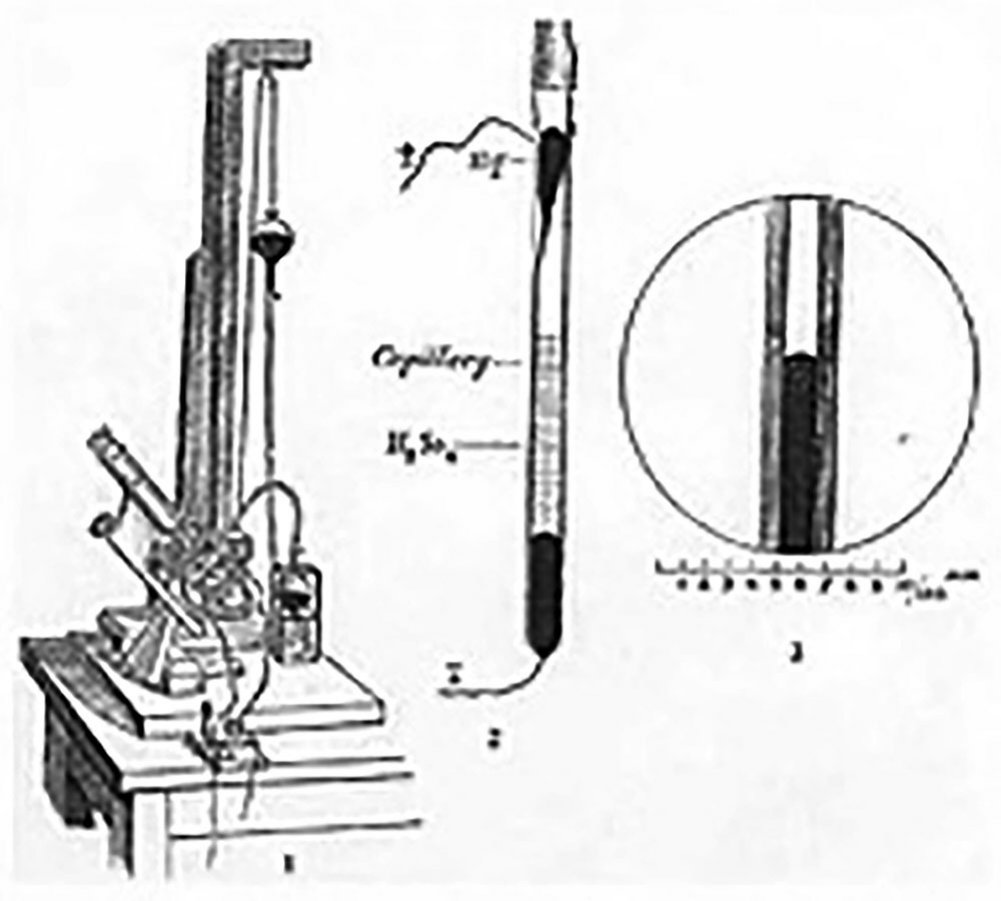
The capillary electrometer detected small electric currents by measuring changes in the surface tension of mercury within a capillary tube. The device, consisting of a mercury‐filled tube with sulfuric acid above it, was crucial in early physiological research and served as the basis for the first practical ECG machine. Image from Wikimedia Commons.

Buchanan employed multiple techniques to measure muscle electrical activity, including the capillary electrometer (Figure 9), which recorded voltage changes, the rheoscope, which detected electrical flow, and the muscle sound transmission method, which used a telephone receiver to identify oscillatory patterns in response to stimulation. These tools allowed her to examine both externally induced and naturally occurring muscle contractions.

One of her primary investigations focused on artificial tetanus, where she stimulated the muscle with electrical pulses at varying frequencies (from 50 to over 500 pulses per second). She observed that high‐frequency stimulation resulted in a relatively steady electrical response, whereas lower frequencies produced oscillatory patterns. This suggested that muscle fibres respond differently depending on the rate of stimulation, a concept later explored in depth through modern electrophysiological studies on tetanic fusion and rate coding.

Buchanan also examined how mechanical tension influenced electrical activity. By incrementally increasing the stretch on the muscle, she demonstrated that greater tension was associated with higher amplitude electrical responses. This was an early indication of the interaction between mechanical loading and electrical properties of muscle, foreshadowing later work on mechanotransduction.

The effects of temperature were another key focus of her study. She showed that cooling the muscle slowed the frequency of oscillatory electrical patterns, while warming accelerated them, an observation that aligned with the known relationship between metabolic activity and muscle excitability.

In her experiments with chemically induced contractions, she investigated electrical activity during strychnine‐induced spasms and veratrine‐induced contractions. She noted distinct rhythmic variations in the electrical response and identified cases where muscle‐generated rhythms were absent, suggesting different mechanisms underlying each type of contraction.

Buchanan also compared voluntary contractions to electrically induced ones, finding that voluntary contractions produced more variable electrical signals, whereas induced contractions resulted in more uniform responses. This distinction contributed to an early understanding of stimulus‐dependent variability in neuromuscular activation.

Although Buchanan did not have access to modern electrophysiological techniques, her systematic approach provided one of the earliest detailed investigations into the electrical behaviour of skeletal muscle. Her work offered valuable insights into how frequency, mechanical conditions, temperature and chemical factors influence muscle excitability, laying the foundation for later research in neuromuscular physiology.

## 8 TRANSITION TO INDEPENDENCE

In 1904, after completing her groundbreaking research on the electrical processes of muscle contraction and earning her doctoral degree, Florence Buchanan faced a turning point in her career. Her mentor, John Scott Burdon‐Sanderson, retired at the age of 75, bringing a pivotal chapter of her professional journey to a close. A steadfast advocate for Buchanan and her work, Burdon‐Sanderson passed away the following year, leaving Buchanan to chart her course independently. Historical records from that same year indicate she was made a fellow of University College London (Nature, 1931) – a remarkable accomplishment for a woman in science at the time. Shortly after, she moved her work to the Oxford University Museum of Natural History, where she set up her own laboratory (Ashcroft, 2020; Creese, 2004). Equipped with essential tools and supported by research grants from the Royal Society (Ashcroft, 2020; Creese, 2004), Buchanan fully embraced this new phase of independence.

Her research interests evolved toward cardiac physiology, with a focus on heart rhythms. She became fascinated by how heart rates varied across species, in states of hibernation, and during physical exertion. One of her notable studies involved comparing heart rates in awake versus hibernating dormice, revealing the physiological adaptations associated with seasonal dormancy (Buchanan, 1912). This new lab marked the start of an era in which Buchanan would delve deeply into the mysteries of the heartbeat, further establishing her legacy as a force in physiology. Yet, even as her studies expanded into the complex rhythms of the heart, Buchanan remained captivated by the electrical responses of skeletal muscle – a line of inquiry that continued to deepen her understanding of the interconnectedness between muscle and nerve. Her 1908 study (discussed in more detail below) exemplified this ongoing commitment, capturing the nuanced electrical dance of voluntary, reflex, and artificial muscle contractions in a way that set the stage for modern physiology.

## 9 NEW PERSPECTIVE ON MUSCLE AND NERVE

Florence Buchanan's 1908 paper, ‘The electrical response of muscle to voluntary, reflex, and artificial stimulation’ (Buchanan, 1908), examined the electrical activity of muscle under different types of activation. At the time, it was widely accepted that the central nervous system dictated muscle activation, but Buchanan's findings suggested that muscles exhibited distinct electrical responses depending on how they were stimulated.

Her study began with frog muscle experiments, where she applied strychnine to induce reflexive contractions. Using a capillary electrometer, she identified two types of oscillatory patterns in the electrical response: high‐frequency ‘wavelets’ and lower‐frequency ‘waves’. By independently altering the temperature of the muscle and spinal cord, she found that the lower‐frequency waves were affected by spinal cord cooling, while the wavelets remained stable. This indicated that some electrical activity originated in the muscle itself rather than solely from neural input.

**FIGURE 10:**
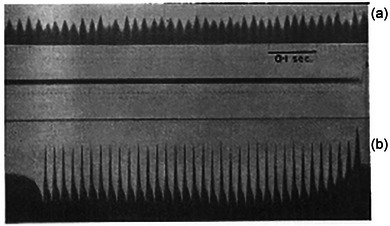
Electrical and mechanical responses of the flexor muscles in the lower arm in a 1908 paper in *Experimental Physiology*. (a) Electrical response of the flexor muscles during voluntary activation, showing a strong and regular pattern with a frequency of approximately 56 Hz. The mechanical force recorded by the dynamometer reached 37 on the scale. (b) Electrical response of the same muscle to artificial stimulation via a series of 35 double induction shocks at a frequency of 50 Hz. While the electrical response remained strong and regular, the mechanical output was dramatically reduced, with the dynamometer registering only 2 on the scale. The recordings also show lingering electrical effects between shocks, a common feature of artificial stimulation techniques used during that era. Reprinted with permission. To the author's knowledge, this experiment represents one of the earliest known instances of electrical stimulation applied to human muscle – a technique that has since become foundational in neuromuscular physiology and clinical practice. Buchanan's innovative work at the turn of the 20th century paved the way for generations of researchers, including contemporary scientists, who continue to rely on this method to investigate muscle function and motor control. It is remarkable to reflect on how her early experiments, conducted well over a century ago, laid the groundwork for techniques still in widespread use today.

She extended her investigation to human muscle, measuring electrical activity in the forearm and jaw muscles during voluntary contractions. These contractions produced irregular and variable oscillatory patterns, differing from the more uniform responses seen in reflexive contractions. When she applied artificial electrical stimulation, the muscle response was more predictable and rhythmic, contrasting with the variability observed in voluntary contractions (Figure 10). These differences suggested that voluntary, reflexive and artificially induced contractions were governed by different mechanisms.

One of Buchanan's key observations was that voluntary contractions did not generate a simple, repetitive electrical pattern. Instead, the signals varied, suggesting a more complex interaction between neural input and muscle response. This work provided early evidence that muscle activation is influenced by factors beyond central nervous system control, including mechanical and physiological conditions.

Although Buchanan did not have access to modern electrophysiological techniques, her findings contributed to an early understanding of muscle electrical activity and its dependence on stimulation type. Her work provided a systematic examination of neuromuscular responses and helped refine the understanding of muscle function at the time.

## 10 UNCREDITED CONTRIBUTIONS

**FIGURE 11:**
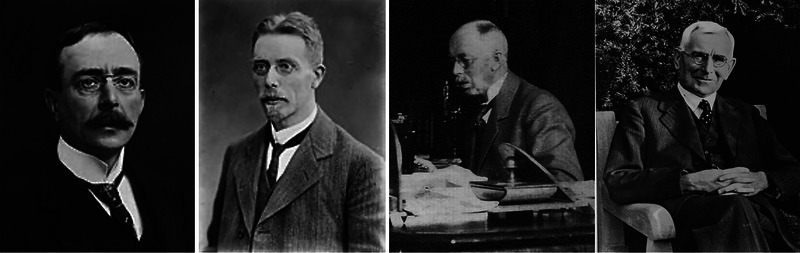
Key collaborators of Florence Buchanan. This four‐panel figure highlights notable scientists who collaborated with Florence Buchanan, showcasing her contributions to the scientific community through interdisciplinary partnerships. From left to right: Sir Charles Scott Sherrington, MD (1857–1952): A pioneer in neurophysiology, known for his work on reflexes and the synaptic transmission of nerve impulses. He was awarded the Nobel Prize in Physiology or Medicine in 1932. Image from Wikimedia Commons. August Krogh, PhD (1874–1949): another Nobel laureate (1920) celebrated for his contributions to physiology, particularly the regulation of capillaries and metabolism. Image from Wikimedia Commons. Johannes Lindhard, MD (1870–1947): a Danish physiologist renowned for his research in exercise and respiratory physiology. Image from Geni.com (https://www.geni.com/photo/view/6000000003232267675?album_type=photos_of_me&photo_id=6000000013383319216). Claude Gordon Douglas, MD (1882–1963): an influential physiologist who advanced the understanding of respiratory gas exchange and exercise physiology. Image from The Royal Society Publishing (https://royalsocietypublishing.org/doi/pdf/10.1098/rsbm.1964.0004).

Despite her groundbreaking contributions, Florence Buchanan's career was often characterized by a duality of collaboration and exclusion. She was undoubtedly a leader in her field, frequently collaborating and engaging in discussions with future Nobel Prize winners Charles Sherrington and August Krogh (Figure 11) (Creese, 2004; Krogh & Lindhard, 1913). In 1910 she received the American Association of Collegiate Alumnae's prize for original scientific research. However, while she drove the field forward and advanced scientific knowledge, she was frequently overlooked when it came to more formal recognition. A notable example of this occurred in her collaboration with August Krogh and Johannes Lindhard (Figure 11) on their 1913 *Journal of Physiology* article investigating the central control mechanisms during exercise in humans (Krogh & Lindhard, 1913). The study examined changes in ventilation, blood flow and pulse rate as subjects transitioned from rest to light or heavy work.

Krogh and Lindhard conducted their experiments in Copenhagen using a custom‐designed bicycle ergometer created by Krogh. While they were able to measure ventilation and blood flow, their methods lacked the ability to record the electrocardiogram, a key metric for understanding heart rate dynamics. Fortunately, Buchanan stepped in to fill this gap. As acknowledged in their paper, ‘Miss Buchanan has shown us the very great kindness to take some electrocardiograms on subjects starting work on a stationary tricycle.’ Her efforts involved collecting data on eight subjects, including Dr C.G. Douglas (Figure 11), the celebrated respiratory physiologist and inventor of the Douglas bag, a device still in use today for collecting respiratory gases.

Buchanan's meticulous data revealed an immediate and rapid increase in heart rate at the onset of exercise, a response that suggested central control rather than reflexive mechanisms – a pivotal insight for the study. Her contributions were indispensable, providing the missing piece that tied together Krogh and Lindhard's findings. However, despite her critical role, Buchanan was not credited as a co‐author.

Krogh, who later won the Nobel Prize and co‐founded the company Nordisk – which merged in 1989 to form the pharmaceutical giant Novo Nordisk – arguably exemplified the systemic inequities of the era by excluding Buchanan's name from the author list. This lack of acknowledgment was emblematic of the challenges faced by women in science during Buchanan's time, where their contributions were often undervalued or erased entirely, even as they played key roles in advancing the field.

## 11 STRUGGLES OF WOMEN IN SCIENCE

Florence Buchanan's struggle for recognition extended beyond her groundbreaking research – it played out in the very institutions that were meant to foster scientific collaboration. Her relationship with The Physiological Society, one of the most prestigious organizations in British physiology, exemplifies the hurdles she faced as a woman in science.

**FIGURE 12:**
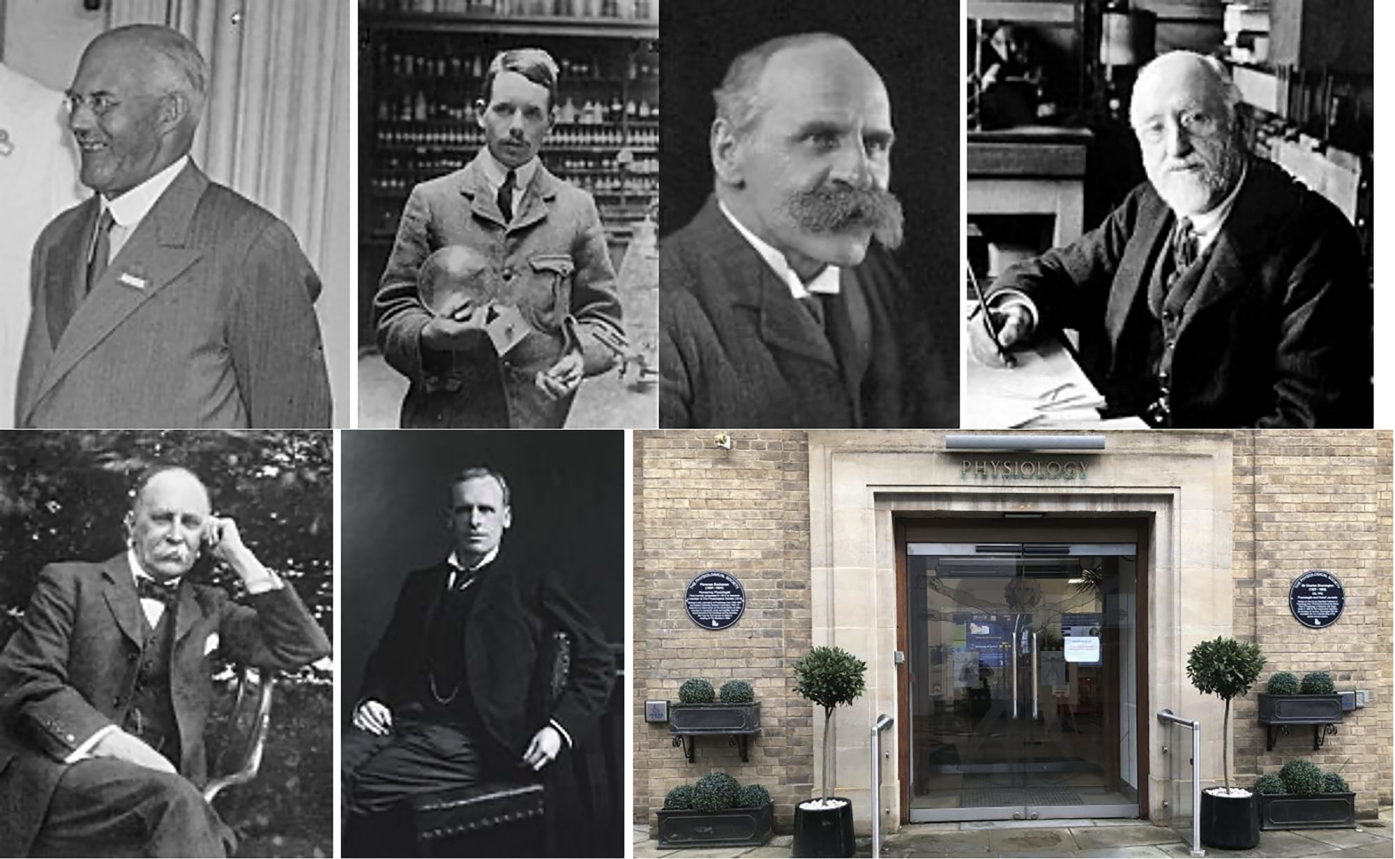
Prominent figures and commemorations associated with Florence Buchanan's career and struggles for recognition. This seven‐panel figure highlights individuals and commemorations linked to Florence Buchanan's journey as a physiologist and her efforts to overcome gender‐based barriers in science. Top row (left to right): Sir Harold Hartley (1878–1972): a chemist and controversial figure in Buchanan's career, known for his initial opposition to her presenting at the Junior Scientific Society of Oxford. Image from Wikimedia Commons. Henry Moseley (1887–1915): a physicist and supporter of Buchanan, who successfully ensured she was allowed to address the Junior Scientific Society by reading a letter of support when opposition arose. Image from Wikimedia Commons. John Scott Haldane (1860–1936): a renowned respiratory physiologist, family friend of Buchanan, and advocate for the inclusion of women in The Physiological Society. Image from Wikimedia Commons. Sir William Maddock Bayliss (1860–1924): a leading vascular physiologist who seconded Haldane's motion to admit women to The Physiological Society. Image from Wikimedia Commons. Bottom row (left to right): Sir William Osler, MD (1849–1919): commonly referred to as the ‘Father of Modern Medicine’ and one of the "Big Four" founding professors of Johns Hopkins Hospital, Sir William Osler was a prominent supporter of Buchanan's recognition by The Physiological Society. Image from Wikimedia Commons. Ernest Henry Starling, MD (1866–1927): a celebrated cardiovascular physiologist, remembered both for his scientific contributions and his opposition to admitting women to The Physiological Society. Image from Wikimedia Commons. Blue Plaques commemorating Florence Buchanan and Sir Charles Sherrington: installed on the Sherrington Building at the University of Oxford, these plaques honour their contributions to physiology. The building now houses the Florence Buchanan Lecture Theatre. Image from University of Oxford Medical Sciences Division. These individuals and commemorations illustrate both the challenges Buchanan faced and the lasting recognition of her scientific achievements and strength in overcoming systemic inequities.

As previously noted, she attended her first meeting of The Physiological Society in 1896, becoming the first woman to present her research at its gatherings, yet she was barred from attending the dinners. More than a decade later, she continued to face obstacles and remained on the margins of key scientific organizations. For example, in 1909, Buchanan was invited to present her findings on heart rhythms to Oxford's Junior Scientific Society – an opportunity that seemed routine but quickly became controversial. Some members were outraged at the notion of a ‘Lady’ addressing their group, with one member, Sir Harold Hartley (Figure 11), who was regarded as having ‘unrivalled powers of persuasion’ (The Royal Society, 2024), openly opposing her presence. According to Henry Moseley's account (Figure 12), Hartley tried to rally support but was left to ‘do his own dirty work’ when his allies refused to act on his behalf (Heilbron, 1974). In the end, he didn't confront Buchanan, who delivered her talk undeterred (Ashcroft, 2020).

This defiance in the face of prejudice reflects Buchanan's strong will, a trait acknowledged even in personal accounts. John Scott Haldane's family, who considered her ‘Aunt Florence’, admired her scientific achievements, though his daughter described her as ‘distinguished, if somewhat difficult’ (Mitchison & Squier, 1995). Haldane's wife, however, championed Buchanan as a feminist and valued her as a close family friend (Mitchison & Squier, 1995). Yet Buchanan's commanding intellect and uncompromising nature did not always endear her to others. Naomi Mitchison, who knew her as ‘Aunt Florence’ during childhood visits to the Burdon‐Sandersons, recalled disliking her unkempt appearance, the hairs on her face, and even her scent—though she suspected Buchanan disapproved of her just as much. Plagued by deteriorating eyesight due to a detached retina (Buchanan, 1908), Buchanan nevertheless maintained her fierce independence and scientific rigor. These complex personal relationships, shaped by both admiration and friction, offer a glimpse into the kind of woman who could challenge the scientific establishment despite the barriers placed in her path.

By 1913, Florence Buchanan had firmly established herself as an independent physiologist with a diverse body of research spanning muscle and cardiac physiology. Her contributions were reflected not only in the 10 presentations she delivered at The Physiological Society meetings but also in her extensive publication record across several respected journals of the time. She published single‐author papers in *The Journal of Physiology* (Buchanan, 1899, 1901) and the inaugural volume of *The Quarterly Journal of Experimental Physiology* (Buchanan, 1908), which later became *Experimental Physiology*, as well as contributions to *The Proceedings of the Royal Society of Medicine* (Buchanan, 1912) and *The Transactions of the Oxford University Scientific Club* (Buchanan, 1909).

Buchanan's research covered a wide range of physiological topics, particularly the electrical and mechanical properties of muscle. In her studies on veratrinized muscle, she examined how exposure to veratrine – an alkaloid known to alter sodium channel function – affected muscle contraction (Buchanan, 1899). Veratrine causes prolonged depolarization of muscle fibres, leading to sustained contractions rather than the brief, transient responses typically seen with electrical stimulation. Buchanan systematically measured both isometric (fixed‐length) and isotonic (variable‐length) contractions to determine how chemically induced excitability altered muscle function. Her work contributed to the broader understanding of excitation–contraction coupling, particularly how pharmacological agents modulate muscle response.

Beyond skeletal muscle, Buchanan also investigated cardiac electrophysiology, focusing on how heart function is altered under different physiological conditions. One of her key studies examined auricular (atrial) and ventricular dissociation – a phenomenon in which the electrical and mechanical activities of the atria and ventricles become uncoupled. Normally, the atria contract first, followed by the ventricles in a coordinated sequence controlled by electrical impulses traveling through the heart's conduction system (Buchanan, 1912). In certain conditions, such as extreme cooling or disease, this coordination can break down, resulting in independent atrial and ventricular contractions. Buchanan studied this phenomenon in hibernating dormice, small rodents that enter a state of torpor during the winter, significantly reducing their metabolic rate and body temperature. By recording electrocardiograms at different stages of hibernation, she observed that as body temperature dropped, ventricular contractions slowed significantly, while atrial activity sometimes continued at a different, faster rate. This suggested that temperature‐induced metabolic suppression altered the normal conduction pathways between the atria and ventricles.

Her findings demonstrated that cardiac conduction is not a fixed process but is influenced by physiological and environmental factors, such as temperature and metabolic state. These observations provided early insights into how hibernating animals regulate heart function under extreme conditions and contributed to the broader understanding of cardiac electrophysiology and conduction disorders.

This body of work highlights the breadth of Buchanan's research and her ability to investigate fundamental physiological processes across multiple organ systems. Her studies contributed to the evolving understanding of muscle excitability, contractile mechanics and cardiac conduction, reinforcing her position as a leading experimental physiologist of her time.

And yet, despite her clear accomplishments, women like Buchanan were still excluded from full membership in The Physiological Society. It was during 1915 that John Scott Haldane (Figure 12), a distinguished respiratory physiologist and close family friend of Buchanan's, formally proposed that women should be eligible for membership in The Physiological Society, a motion underscored by the legacy of his maternal uncle, John Burdon‐Sanderson, who had mentored and advocated for Buchanan early in her career (Ashcroft, 2020; Groll, 2015). Haldane's motion was seconded by William Bayliss (who had also studied under Burdon‐Sanderson) (Figure 12), a renowned vascular physiologist at University College London, as well as other notable figures, such as William Osler (the ‘father of modern medicine’) (Figure 12) and future Nobel laureate Charles Sherrington (Ashcroft, 2020; Groll, 2015). These men clearly recognized Buchanan's immense contributions and sought to rectify the inequities of the Society's policies.

However, their proposal faced fierce opposition from some of the society's most prominent figures (Groll, 2015). Among them was Ernest Henry Starling (Figure 12), a legendary cardiovascular physiologist who was nominated for the Nobel Prize four times to no avail, known for formulating the ‘law of the heart’ and the ‘law of the capillaries’ (Henriksen, 2005). Starling dismissed the idea of admitting women by quipping that the Society was primarily a ‘dining organization’ and that it would be improper to dine with ‘ladies smelling of dog’ – though, he clarified, it was the men who smelled of dog after long hours in the lab (Groll, 2015). Of interest, he and Bayliss, a strong proponent for Buchanan's admission, were brothers‐in‐law. His remark – whether intended to be lighthearted or not – captures the entrenched biases women like Buchanan faced, where their presence was seen as a disruption to the established norms of male‐dominated spaces.

For 2 years, Starling and his allies successfully blocked the motion (Groll, 2015). It was not until January 1915 that the Society finally voted to admit women as full members (Groll, 2015). By then, Buchanan had spent nearly two decades contributing to the Society's meetings without being allowed to join its most vital social rituals. At last, she and five other trailblazing women became the first female members of The Physiological Society (Groll, 2015). Buchanan could now attend the same dinners she had long been excluded from.

The admission of women to The Physiological Society marked a significant step forward, but it also highlighted how deeply ingrained the barriers to inclusion were, even for someone as accomplished as Florence Buchanan. Her perseverance, in the face of these systemic inequities, speaks to her character as much as her scientific brilliance. Buchanan's story serves as a reminder that progress in science has often required more than intellect – it has demanded persistence, determination and the courage to keep moving forward when doors remain stubbornly closed.

## 12 LASTING LEGACY

Florence Buchanan's life was marked by perseverance, intellect and an unrelenting tenacity that propelled her to continue working despite the many challenges she faced. Her research on muscle and heart physiology created a foundation for modern neuromuscular and cardiac studies, leaving an indelible mark on the field. Even as her eyesight began to fail in her later years (Nature, 1931), Buchanan remained steadfast in her dedication to science, continuing her work at the University Laboratory of Physiology for as long as she was able.

**FIGURE 13:**
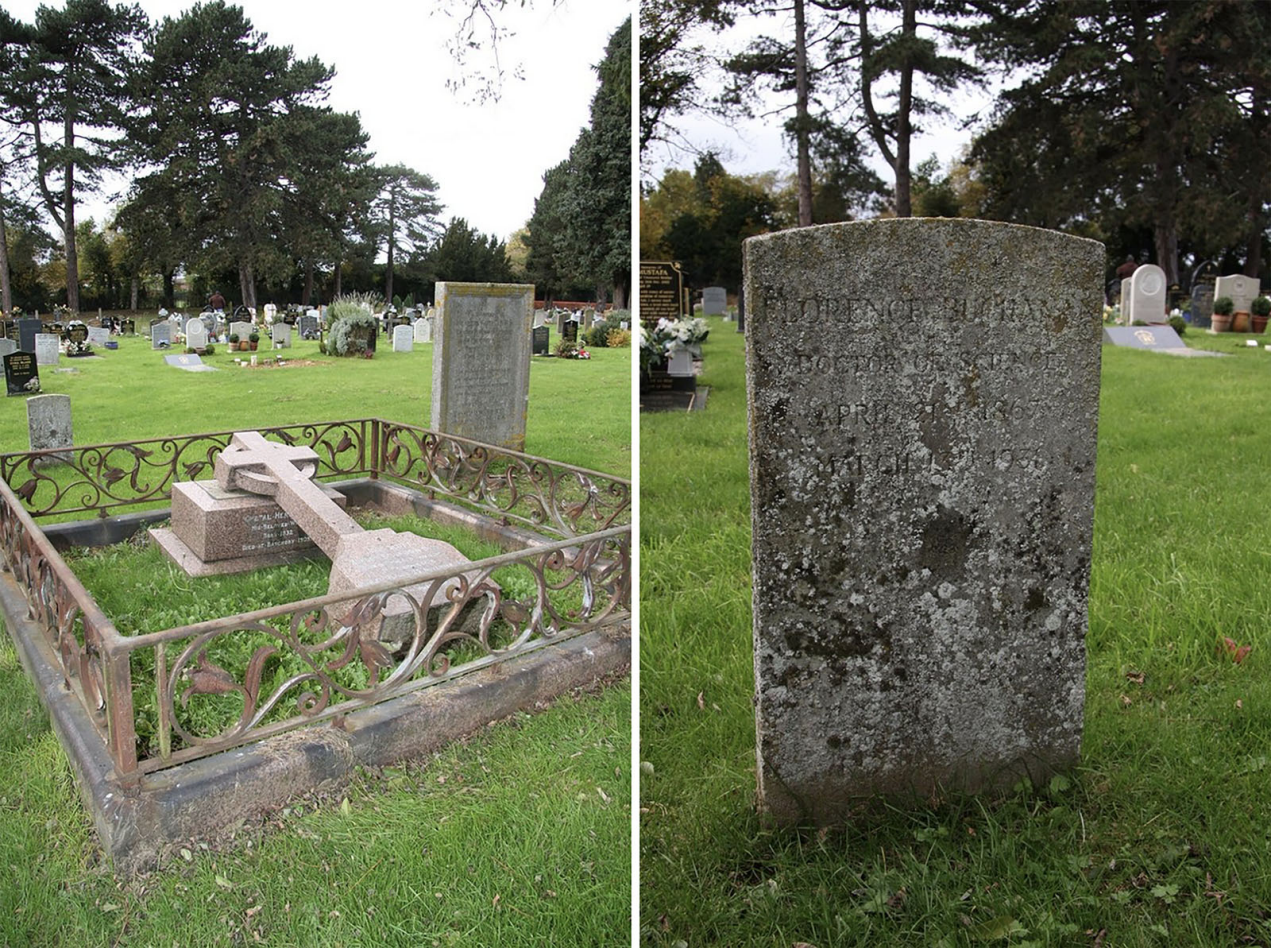
Final resting places of Florence Buchanan and John Burdon‐Sanderson. The image on the left shows the grave of John Burdon‐Sanderson, a close family friend of Florence Buchanan and an early mentor and advocate for her career in physiology. His grave is marked by a large headstone and enclosed by a decorative iron fence. To the rear and slightly left of Burdon‐Sanderson's grave is the modest headstone marking Buchanan's final resting place, which leans slightly, reflecting the passage of time. The image on the right provides a closer view of Buchanan's headstone, which is simple and inscribed: ‘Florence Buchanan, doctor of science, April 21st, 1867, March 13th, 1931.’ The proximity of their graves symbolizes their enduring connection and Burdon‐Sanderson's influence on Buchanan's scientific career. Images from findagrave.com. (Left: https://www.findagrave.com/memorial/60923703/john‐scott‐burdon‐sanderson/photo#view‐photo=35013642; right: https://www.findagrave.com/memorial/60933962/florence‐buchanan#view‐photo=35014297).

Florence Buchanan passed away on 13 March 1931, at the age of 64. She was laid to rest in Wolvercote Cemetery in Oxford, her grave placed near that of her mentor, Burdon‐Sanderson, whose guidance had played a pivotal role in the early years of her career (Figure 13). The absence of any known photographs of Buchanan serves as a poignant reminder of how easily history can overlook the contributions of women in science, even as their male counterparts are immortalized through images and tributes. Yet her legacy endures in the work she left behind and the barriers she helped to break.

As a white male reflecting on Buchanan's life, I am struck by how often women's contributions are overshadowed, including those who have shaped my own career. My wife, with degrees in engineering mechanics and a doctorate in physical therapy, has been a key collaborator in my lab, her insights shaping my work in immeasurable ways. Likewise, my PhD advisor played a pivotal role in my development, teaching me both critical thinking and the determination needed to succeed in this field. These experiences have deepened my appreciation for the challenges women face and the brilliance they bring to science.

These personal experiences remind me that Florence Buchanan's struggles are not relics of the past. The barriers she faced, though diminished, are not entirely gone, and her story challenges us to reflect on the progress still needed. Her legacy is more than a testament to the courage it takes to push the frontiers of knowledge in the face of exclusion; it is also a call to action. Her life invites us to celebrate those who have been historically overlooked and to challenge the systems that still perpetuate inequity today. Florence Buchanan's resilience and brilliance light the way forward – both as an enduring inspiration and as a guide for how we might strive to do better.

## AUTHOR CONTRIBUTIONS

All authors have approved the final version of the manuscript and agree to be accountable for all aspects of the work in ensuring that questions related to the accuracy or integrity of any part of the work are appropriately investigated and resolved. All persons designated as authors qualify for authorship, and all those who qualify for authorship are listed.

## CONFLICT OF INTEREST

The author does not have any competing interests with relevance to this article.
